# Prolactin gene diversity and its influence on milk yield traits in Ayrshiredairy cattle from Southern Russia

**DOI:** 10.5455/javar.2025.l973

**Published:** 2025-12-25

**Authors:** Nadezhda Vasilievna Shirokova, Vladimir Khristoforovich Fiodorov, Ivan Fiodorovich Gorlov, Marina Ivanovna Slozhenkina, Elena Yurievna Anisimova, Natalia Ivanovna Mosolova

**Affiliations:** 1 Department of Biotechnology, Don State Agrarian University, Rostov-on-Don, Russian Federation; 2 Department of Biology, Morphology and Virology, Don State Agrarian University, Rostov-on-Don, Russian Federation; 3 Livestock Production Department, Povolzhsky Research Institute of Manufacture and Processing of Meat and Dairy Products, Volgograd, Russian Federation; 4 Department of Livestock Products Storage and Processing, Povolzhsky Research Institute of Manufacture and Processing of Meat and Dairy Products, Volgograd, Russian Federation; 5 Farm Animal Breeding and Genetics Laboratory, Povolzhsky Research Institute of Manufacture and Processing of Meat and Dairy Products, Volgograd, Russian Federation; 6 Dairy Quality Control Laboratory, Povolzhsky Research Institute of Manufacture and Processing of Meat and Dairy Products, Volgograd, Russian Federation

## Abstract

**Objective:**

The objective of this research was to discover single-nucleotide variations in the ­prolactin* (PRL)* gene and to evaluate their correlation with milk production characteristics in Ayrshire cows reared in southern Russia.

**Materials and Methods:**

DNA (deoxyribonucleic acid)samples were extracted from the blood of cows (*n* = 300) using a commercial kit. The polymerase chain reaction-restriction fragment length polymorphism (PCR-RFLP) technique was employed to identify and quantify genotypes and allele frequencies. Genetic indices were calculated, and statistical processing was performed. A general linear model was used to investigate the relationship between single-nucleotide polymorphisms and milk productivity in cows.

**Results:**

The distribution of the AA, AB, and BB genotypes in the studied population was 90%, 0%, and 10%, respectively. The genetic equilibrium in the Ayrshire cow population was preserved (*χ*² = 0.2879). The results indicate the presence of allelic diversity in the prolactin gene in this ­population. The milk yield per lactation in cows with the PRL_AA genotype was 7,378.50 kg, and for PRL_BB, it was 6,569.63 kg. Ayrshire cows with the PRL_BB genotype yielded less milk compared to their PRL_AA counterparts by 10.96% (*p* < 0.05).

**Conclusion:**

Using the obtained results in the future selection of offspring with desirable PRL ­genotypes at an earlier age could significantly increase the efficiency of breeding work in the studpulation and accelerate the process of creating a herd with higher productivity potential.

## Introduction

Currently, the Russian Federation places significant emphasis on developing dairy cattle farming. The agricultural sector faces the challenge of increasing the overall cattle population, developing new breeds, and improving existing ones to create highly productive animals.

The introduction of genetic advancements into selective breeding practices has become particularly important, as they enable the evaluation of animals at the genetic level [1].

Traditional methods based on pedigree analysis and phenotypic data have certain limitations. For example, phenotypic traits related to productivity may only become apparent with age, making early assessment of an animal’s genetic potential challenging. By using DNA markers in animal husbandry, it is possible to identify genes in animals that carry desirable phenotypic traits [2]. The Ayrshire breed is distinguished by high milk yields and rich milk fat content [3]. Russian Ayrshire cattle have a breeding history spanning more than 200 years [4]. Ayrshire cows are highly productive, with a milk yield of 6,400 kg, containing 4.1% fat and 3.3% protein (in average) [5].

Milk productivity is a complex trait controlled by a large number of genetic loci [6].

Prolactin is a hormone that is essential for the process of milk production. It is involved in every step of the process, from the transcription of the milk protein genes to the stabilization of mRNA (messenger ribonucleic acid), translation, and post-translational modifications. Prolactin also plays an important role in immune functions, cellular differentiation, and growth [7,8].

The prolactin gene in cattle includes five exons and four introns and is located on chromosome 23 [9]. Evaluation of the cDNA sequence of the *bPRL* gene in four distinct clones revealed seven potential nucleotide mutations. One of these is a silent A-G substitution in exon III, which occurs in the codon of the 103rd amino acid and results in the presence of a polymorphic RsaI site [10]. Digestion products size for AA, AB, and BB genotypes is 156 bp; 156, 82, and 74 bp; 82 and 74 bp, respectively [11].

By promoting and maintaining lactation, prolactin can serve as a genetic indicator of milk production level, as well as an additional selection criterion in dairy cattle breeding [12]. Nevertheless, the information available in scientific literature regarding the relationship between PRL genotypes and milk production remains contradictory [13].

Therefore, studying the Ayrshire cow population for prolactin gene polymorphism is relevant. Furthermore, there is a lack of knowledge regarding the identification of prolactin(*PRL*) gene diversity and its impact on dairy performance in Ayrshire cows raised in southern Russia.

In this case, our study aimed to discover single-nucleotide variations in the *PRL* gene and to evaluate their correlation with milk production characteristics in the Ayrshire cows’ population from southern Russia. This is the first report of *PRL* polymorphism in Ayrshire cattle from this region.

## Materials and Methods

### Ethical approval

This study was conducted from September 3, 2022, to June 20, 2024, at the Lenin Agricultural Enterprise, Rostov Region, Russian Federation (47°38’45.3”N; 42°03’28.6”E), with ethical approval from the Don State Agrarian University Institutional Review Board (EA DSAU #2-2022-09-01). All procedures complied with EU regulations for the protection of animals used for scientific purposes, and efforts were made to minimize animal discomfort. Permission for the inclusion of cows was granted by the farm owner.

### Animals and housing

**Figure 1.**
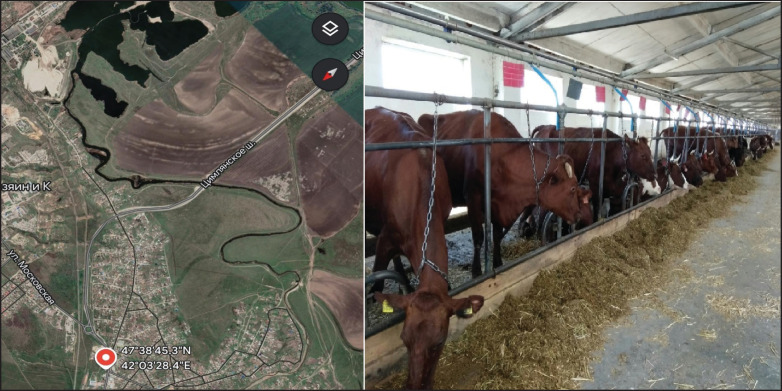
Localization of the Ayrshire breeding reproducer farm and some first-calf cows from the studied population.

All the studied Ayrshire first-calf cows (Fig. 1) were clinically healthy and maintained in optimal conditions, in accordance with zootechnical standards and hygienic requirements.

The experience was performed in the Tsimlyansky district, a major industrial center in the eastern part of Rostov Oblast. The breeding farm is located in a moderately continental climate zone, characterized by insufficient precipitation, dry summers, and long, cold winters, with frequent droughts in the area. This territory lies within steppe and dry steppe landscape types, creating conditions conducive to cattle breeding.

To conduct this research, primary breeding records and the results of the researchers’ evaluations were analyzed, including assessments of parental productivity traits, selection, and laboratory analysis of biological materials. All parent pairs belonged to the Ayrshire breed. Reproduction was organized under conditions of panmixia, which eliminated the effects of directed parental selection. In the experimental group, the age difference among young animals did not exceed four days. Full siblings were excluded from the experimental group.

The farm’s breeding program is focused on enhancing the genetic potential of dairy cattle. The animals are kept under similar conditions and receive a carefully balanced diet, which helps ensure that the productive traits of cows are more likely to be influenced by specific gene alleles.

### Sample collection

Peripheral blood was drawn from the jugular vein into 9.0 ml Vacuette vacuum EDTA-K3 tubes (Greiner Bio-One, Austria) during the assessment of animals (*n* = 300). Cow’s milk yield was determined based on monthly test milking following the rules for assessing milk productivity during lactation. The milk fat content as well as milk protein content were measured using the milk analyzer “MilkoScan FT1” (Foss Electric, Denmark).

### DNA extraction

DNA was isolated from blood samples using the manufacturer’s protocols with the Extran-2 Set (Sintol, Russia) and the Cleanup Mini kit (Evrogen, Russia) for DNA purification. The QuantiFluor(R) dsDNA System E2670 kit for quantitative determination of double-stranded DNA in QuantiFluor(R) dsDNA solution (Biom Group, Russia) was used with Quantus Fluorimeter (Promega, USA), and the DNA concentrations obtained from the samples were 100–200 ng/µl.

### PCR amplification

To amplify a PCR product (156 bp), DNA was extracted, a reaction mix (Table 1) was used, and primers (Sintol, Russia) were employed. Amplification was performed using an ANK-32 thermal cycler (Sintol, Russia). In Table 2, the PCR conditions are presented. Then, PCR samples were stored at 10°C until RFLP Analysis.

**Table 1.** Reaction Mix for PCR Amplification.

Item	Volume, µl
1 × Taq Buffer (Mg_2_+ 1 mM)	1.5
dNTP (0.2 mM)	1.5
HS Taq DNA Polymerase (1 U)	0.08
Forward primer (10 pmol): 5'-CGA-GTC-CTT-ATG-AGC-TTG-ATT-CTT-3'	0.2
Reverse primer (10 pmol): 5'-GCC-TTC-CAG-AGG-TCG-TTT-GTT-TTC-3'	0.2
ddH_2_O	10.52
DNA (50–500 ng)	1
Total volume	15

**Table 2.** PCR conditions.

Stage	Temperature	Time
Primary denaturation	95°C	5 min
Then 30 cycles:		
Denaturation	95°C	30 sec
Annealing	59°C	45sec
Elongation	72°C	1 min
Final extension	72°C	10 min

The identification of the PCR product was carried out using the 10-line (50–700 bp) DNA Ladder 50+ bp (Evrogen, Russia) by electrophoresis in a 2% agarose gel stained with EtBr at 85 V for 60 min, using the appropriate kit (DNA-Technology, Russia). The obtained results were visualized using the gel documentation system GenoSens 2150 (Clinx Science Instruments Co., Ltd., China).

### RFLP analysis

RFLP analysis was performed by digesting in a reaction mixture final volume of 20 µl, containing RsaI restriction enzyme—0.5 µl (10 U/µl), with 10X SE-buffer B—2.0 µl (SibEnzyme, Russia), PCR product—5.0 µl, nuclease-free water—12.5 µl at 37°C for 16 h. The polymerase chain reaction-restriction fragment length polymorphism fragments were identified by electrophoresis (2% agarose gel stained with EtBr, at 85 V for 60 min) compared with 10-line (50–700 bp) DNA Ladder 50+ bp (Evrogen, Russia), and the obtained results were visualized using the GenoSens 2,150 gel documentation system (Clinx Science Instruments Co., Ltd., China).

### Statistical analysis

Genetic parameters were calculated in PopGen 1.32 and Arlequin 3.5.2.2 software [14]; Statistica 10.0 software was used for statistical processing (Statsoft Inc., USA) [15]. The findings were statistically significant at a level of *p* < 0.05.

Association analysis between single-nucleotide polymorphism (SNPs) and the milk production traits was based on the General Linear Model (GLM) procedure using the following statistical model:

*Y*_ij_= *m* + *G*_i_ + *e*_ij’_

where *Y*_ij_ is the observed milk productivity of each animal (a dependent variable; the result, which we are studying: the link between *i* ^th^ genotype and *j* ^th^ milk production trait) with *i* ^th^ genotype for *j* ^th^ cases; *m* is the overall mean (the average productivity value of the entire studied population with all genotypes); *G*_i_ is the fixed effect of the *i* ^th^ genotype (if we have three genotypes AA, AB, BB, then each of them will have its own fixed effect: *G*_1_, *G*_2_, *G*_3_); and *e*_ij_ is the residual error (individual productivity deviations that are not explained by the genotype or the overall mean, but may occur due to factors that were not taken into account: for example, animal health, measurement errors, and so on).

This model is correct for analyzing associations between SNPs and dairy productivity traits if the following conditions are met (key limitations of using the statistical model):

1. The data are consistent with GLM assumptions:
  1.1. Normality of the distribution of residues (*e*_ij_ ),
  1.2. Homogeneity of variances (absence of heteroscedasticity),
  1.3. Linear relationship between predictor and response.
2. The genotype is considered as a fixed effect, which is justified if specific alleles (AA, AB, and BB) are analyzed without generalization to the population.
3. Possible confounding factors are considered (for example, age, lactation, diet, and environmental conditions). If they are not included in the model, this can lead to false associations.

Note that the studied population was selected considering the homogeneity of the cows by age, lactation, diets, and environmental conditions. In this regard, the covariances mentioned above were not included in the statistical model. Thus, in other populations (having heterozygous genotypes, other environmental conditions, and so on), the obtained results may differ from those presented in this article.

## Results and Discussion

### Genetic parameters of studied population

**Figure 2.**
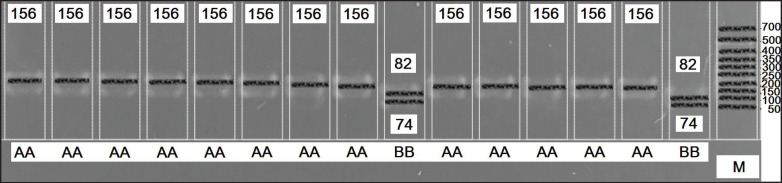
Electropherogram of PRL/RsaI gene: AA genotype—156 bp; BB genotype—82 and 74 bp; M—molecular weight marker.

The electrophoresis results are shown in Figure 2. Both A and B alleles of the *PRL* gene were identified by RFLP analysis with RsaI endonuclease in the studied population (0.90 and 0.10, respectively). However, only homozygosity genotypes were found (PRL_AA—90%, PRL_BB—10%). Allele and genotype distributions are presented in Table 3. The absence of heterozygotes in the studied population of Ayrshire cows is explained by its genetic homogeneity: the selection strategies employed by permanent crosses and planned line rotations can result in a buildup of parental genes and a decline in genetic diversity.

**Table 3.** Frequencies of *PRL*gene alleles and genotypes in Ayrshire dairy cattle (*n*= 300).

Gene	Allele frequency		Genotype frequency					
*PRL*	А	В	АА		АВ		ВВ	
			*n*	%	*n*	%	*n*	%
	0.90	0.10	270	90	0	0	30	10

*n* = number of animals

To assess selective significance, the obtained genotype frequencies were compared to those expected under the Hardy–Weinberg equilibrium. The χ² test for the Ayrshire population was conducted with a significance level of *p* < 0.01. In Table 4, the observed and expected heterozygosity levels of the studied population are presented.

**Table 4.** Heterozygosity levels of the studied Ayrshire dairy cattle population (*n* = 300).

Gene	Na	Ne	Ho	He	F_IS_	χ^2^
*PRL*	1	1.45	0.000	0.312	0.088	0.2879

Our findings on the *PRL* gene genotypes and allele frequencies generally align with those of other researchers. A high frequency of the PRL_A allele is often observed in dairy cattle breeds, ranging from 0.524 to 1.00 [10,16,17].

For example, Argentinian studies of six local cattle breeds (Argentine, Patagonian, “Saavedreño”, “Chaqueño Boliviano”, “Yacumeño”, and “Chusco”) reported Yacumeño breed as monomorphic for this allele, and in the other five frequency was between 0.816 and 1.00 [18]. Russian researchers have identified that Buryat and Altai cattle breeds have similar higher frequencies of the A allele: 0.620 and 0.814 [19].

In contrast, Agrawal et al. [20] found GG and AGwith a complete absence of genotypic pattern AA in three *Bos indicus* cattle breeds (Rathi, Sahiwal, and Kankrej). So, those breeds have lower frequencies of the A allele: 0.36, 0.41, and 0.32, respectively (Bikaner, India).

At the same time, in another Sahiwal cattle population (Karnal, India), the frequencies of the A and G alleles were nearly equal, with a genotypic distribution of 30%, 45%, and 25% for the AA, GG, and AG variations, respectively [21]. Similar results were obtained in Romanian cattle populations: 0.235 and 0.765 for the A and G alleles; 0.10, 0.27, and 0.63 for the AA, AG, and GG genotypes, respectively [22].

Although in Russian Red Pied Cattle was found three genotypes of *PRL* gene (0.598, 0.392, and 0.010 for AA, AB, and BB, respectively), nevertheless, the frequency of the A allele was higher than B (0.794 and 0.206, respectively) [23]. Similar results were found in the Indian population of Gaolao [24] and Sahiwal cattle [25], as well as in the Turkey population of Holstein cows: three genotypes of *PRL* gene and predominance of A allele [11,26,27]. However, in four native Turkish cattle breeds (South Anatolian Red, Turkish Grey, East Anatolian Red, and Anatolian Black), two genotypes of *PRL* gene were found, but there were more heterozygous genotypes: AA—0.400, AB—0.600 [10]. Herewith, Gayari et al. [28] identified three genotypes for *PRL* gene in Indian population of Jersey × Red Sindhi crossbred cows, along with that more often an AB genotype was found, then AA and then BB.

The reduced genetic diversity, for example, in an isolated population, will unavoidably increase in breeding. Similarly, the allelic diversity of genes subjected to strong selection pressure can lead to a significant deviation in the frequency of alleles in one direction or another. This may explain the high frequency of allele A that has been found in the studied population.

The high frequency of the A allele in Indian cattle, as reported by Bangar et al. [12], suggests that current breeding efforts are favoring the spread of that allele in the population.

### Dairy productivity and milk quality by PRL genotype

Average milk yield (AMY), fat (FC), and protein (PC) content in milk, and their amounts per lactation (FY, PY) in Ayrshire first-calf cows with different *PRL* gene genotypes were evaluated (Table 5).

**Table 5.** Milk productivity of Ayrshire cows with different *PRL*gene genotypes.

Parameter	Genotype, *n*	
	AA, 270	BB, 30
Milk yield, kg	7378.50 ± 238.31^a^	6569.63 ± 312.52^b^
Milk fat content, %	4.15 ± 0.09^a^	4.37 ± 0.06^b^
Milk fat yield, kg	306.21 ± 20.32^a^	287.09 ± 10.98^a^
Milk protein content, %	3.45 ± 0.07^a^	3.53 ± 0.04^a^
Milk protein yield, kg	254.56 ± 13.45^a^	231.91 ± 12.81^a^

*n* = number of animals; Data are reported as mean ± SEM; Means in row followed by different superscript letters are significant at *p* < 0.05.

Ayrshire cows with the PRL_BB genotype produced less milk than their PRL_AA counterparts by 808.9 kg (*p* < 0.05). Hence, the milk fat content in PRL_AA cows has been less by 0.22% than in PRL_BB cow’s milk (*p* < 0.05). The PRL_AA genotype had also less protein content in milk than the PRL_BB genotype by 0.08%, but with no significance. There also were no significant differences in milk fat and milk protein yields per lactation between groups.

In the literature data about the association of *PRL* genotypes with dairy productivity also remain inconsistent [1,9,12,13,22,24]. For example, Alipanah et al. [29] found that in milk from Black Pied cows with the AA genotype, protein content and milk yield were higher than in BB genotype (by 0.1 and 6%, respectively); however, in BB genotype milk was higher fat content (by 0.19%). In Red Pied cows BB genotype was positively associated with milk yield (by 11.1%) but had less fat percentage in milk (by 0.08%) and had no association with milk protein content.

Rincón et al. [30] reported that in Colombian dairy herds of Holsteins, the polymorphism of the prolactin (*PRL*) gene did not significantly affect any of the measured parameters. However, the effect of the AA variation on milk yield was nearly significant. Karuthadurai et al. [21] discovered that the G55A SNP was linked to milk production characteristics and could be used as a potential genetic indicator for selecting young Sahiwal cattle.

At the same time, Agrawal et al. [20] found that cows of the Rathi, Sahiwal, and Kankrej breeds with heterozygous genotypes produce milk with a higher protein content (by 0.16%) compared to cows with the PRL GG genotype.

A comprehensive analysis spanning the years 2002 to 2022 [1] revealed that variations in the *PRL* gene were linked to milk production, fat content, and protein yield in Holstein cattle; the PRL_AB genotype also demonstrated superior performance compared to other genotypes.

Through the implementation of the linear model, Fang et al. [2] conducted association analyses based on SNPs and haplotype blocks, which allowed them to identify the genetic influence of *PRL* on milk production characteristics in Chinese Holstein cattle.

Bangar et al. [12] in the meta-analysis (2005–2020) revealed that AB genotype had the highest impact on productive traits in Indian dairy cow herds, followed by the AA genotype and then the BB genotype [28]. In contrast, Varay et al. [25] reported that Sahiwal cows with AA genotypes had higher milking than AB and BB.

Therefore,* PRL* gene polymorphisms could be used as good markers for enhancing milk production and are still insufficiently studied. The benefit of genetic markers is that they are unaffected by environmental factors, follow a specific pattern of inheritance, and can be precisely controlled. However, it is important to note that in our study, results only about the *PRL* gene polymorphism and its association with AMY, FC, PC, FY, and PY, exactly for this population, were obtained, which opens up opportunities for further research.

## Conclusion

The first results of prolactin gene polymorphism and genetic structure of the Ayrshire dairy cattle population from southern Russia were obtained (*n* = 300). A high frequency of the A allele was exhibited (0.9); moreover, 90% of the studied population had PRL_AAgenotype. Heterozygous PRL_AB animals were not identified. The absence of heterozygotes in this population of Ayrshire cows is explained by its genetic homogeneity. Results also showed that in the studied population of Ayrshire first-calf cows, the AA genotype was linked to variations in milk yield (*p* < 0.05). Nevertheless, in milk of animals with the BB genotype was found more fat content (*p* < 0.05) as well as the protein content (but with no significance). Consequently, the B allele may be favored to enhance the FC and PC; however, the decreasing of AMY. These findings support selection efforts aimed at identifying genetic markers linked to milk productivity, enabling the targeted selection of animals with high genetic potential based on breed and population characteristics. Using the obtained results in the future selection of offspring with desirable PRL genotypes at an earlier age could significantly increase the efficiency of breeding work in the studied population and accelerate the process of creating a herd with higher productivity potential.

## List of abbreviations

DNA, deoxyribonucleic acid; PCR-RFLP, polymerase chain reaction-restriction fragment length polymorphism; *PRL*, prolactin gene; SNP, single-nucleotide polymorphism.
